# The Effect of Material Microstructures on Tool Edge Preparation of PCBN Cutting Tools

**DOI:** 10.3390/mi17050630

**Published:** 2026-05-21

**Authors:** Zhiping Huang, Xian Wu, Chao Zhang, Yanxin Zhai

**Affiliations:** 1 Research Institute of Areo-Engine, Beihang University, Beijing 102206, China; zhaiyanxin@buaa.edu.cn; 2 College of Mechanical Engineering and Automation, Huaqiao University, Xiamen 361021, China; 22014080109@stu.hqu.edu.cn

**Keywords:** tool chamfer grinding, tool edge blunting, PCBN tool

## Abstract

PCBN tools are widely used in the machining of ferrous metals. Tool edge preparation is a crucial procedure in the tool preparation process that directly affects tool performance. In this paper, tool chamfer grinding and edge blunting were conducted on the PCBN tool to investigate the effect of material microstructures. In tool chamfer grinding, the PCBN tool with larger particles exhibits a larger chamfer width error and roughness than that of smaller particles, and the PCBN tool with higher Al content exhibits a larger chamfer width error and roughness than that with lower Al content. The optimal tool chamfer grinding speed is 24 m/s for the PCBN tool with larger particles, and 27 m/s for smaller particles. The optimal feed rate is 70 mm/min for both PCBN materials. In edge blunting, PCBN tools with larger particles or lower Al content are more difficult to passivate, and the optimal blunting time is about 30 s for an edge radius of 30 μm. The PCBN tools were prepared using the obtained machining parameters and used in the turning of brake pads. It is found that the PCBN tool with smaller particles exhibits longer life than that of larger particles. Although it exhibits the same wear characteristics, the tool life of the PCBN tool with lower Al content is longer than that of the tool with higher Al content.

## 1. Introduction

Polycrystalline cubic boron nitride (PCBN) is prepared by high-temperature and high-pressure sintering of cubic boron nitride (CBN) grains and an adhesive phase. It exhibits the second-highest hardness in tool materials, and has excellent chemical inertness and thermal stability at high temperatures [1,2,3]. These dominant advantages make PCBN the ideal material to create tools for ferrous metals [4,5]. At present, PCBN tools are widely used in machining various ferrous metals, such as hardened steel, high-manganese steel, and cast iron [6,7]. The main application fields of PCBN tools include automobiles, heavy machinery, bearings, gears, etc. Among these, automobiles are the largest market for PCBN tools. In terms of the machining process, PCBN tools are mainly used for the turning process, accounting for approximately 60%, followed by the boring process, which accounts for about 30%.

Although PCBN tools are the most suitable for machining cast iron materials, their geometric parameters have an important effect on tool performance [8,9]. Each workpiece’s materials and machining conditions has its corresponding most suitable tool geometric structure, especially the geometric parameters of the tool edge. The purpose of tool design based on simulation or physical experiments is to obtain the optimal tool geometric structure [10,11]. Wu [12,13] established a cutting simulation for the structure design of PCBN tools and obtained the optimal tool geometric structure for the machining of cast iron material. Chen [14] compared the tool wear of PCBN tools with either a variable or an invariable chamfer edge, and found that the PCBN tool with a variable chamfer edge exhibits less tool wear. Zhang [15] studied the effect of the edge radius of PCBN tools in the machining of Zr-based bulk metallic glasses and found that adhesive and oxidation wear were the main wear mechanisms.

The preparation of PCBN tools for the turning process includes the welded type and the integral type. After the tool design, there are three main procedures in the preparation of an integral PCBN tool: tool blank forming, face grinding, and edge preparation [16,17]. Zhang [18] investigated the face grinding of PCBN materials and obtained the optimal machining parameters for PCBN tools. Müller [19] proposed a force-compliant grinding method for PCBN tools that considers the thermal and mechanical loads and the productivity. Denkena [20] studied the grinding of an S-shape PCBN tool and found that the contact length greatly affects the wear mechanism. Among the three procedures of PCBN tool preparation, tool edge preparation is the most crucial procedure that directly affects tool performance. However, there is still less literature focused on the tool edge preparation of PCBN tools, especially when considering the PCBN materials’ microstructures; this limits the full potential of PCBN tools.

In this paper, the tool edge preparation of PCBN tools is investigated, including the preparation of the tool’s chamfer and edge radius. The effect of PCBN material microstructures, such as grain size and adhesive phase, on the tool edge preparation is deeply investigated. Simultaneously, the grinding parameters for the tool chamfer are optimized by comprehensively considering the tool chamfer error and surface roughness, and the optimal blunting parameter for edge round is also analyzed. Finally, the prepared PCBN tool is applied in the turning of brake pads to verify the tool’s performance and investigate the effect of material microstructures on tool performance.

## 2. Materials and Methods

**Figure 1:**
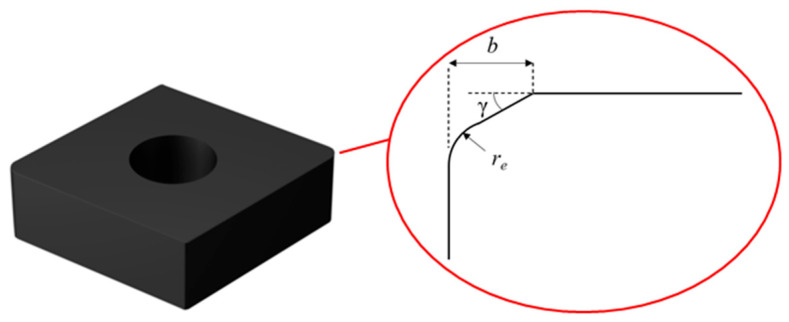
The tool edge parameters of the PCBN tool.

To study tool edge preparation, a PCBN tool blank with the grade of CNMN120412 was formed, as depicted in Figure 1. This is a widely used tool grade in the machining of brake pads and other axis parts. This PCBN tool has a tool tip wedge angle of 80°, rake and relief angles of 0°, an edge length of 12 mm, a tool thickness of 4 mm, and a tool tip radius of 1.2 mm. The tool cutting edge dimensional parameters are crucial to obtain better tool performance, which usually includes the chamfered width *b*, the chamfered angle γ, and the tool edge radius *r_e_*. In this work, the chamfered width and angle are, respectively, set to 0.1 mm and 15°, and the cutting edge radius is set to 30 μm, according to previous research [13]. The objective of tool edge preparation is to realize the machining of the aforementioned parameters.

**Figure 2:**
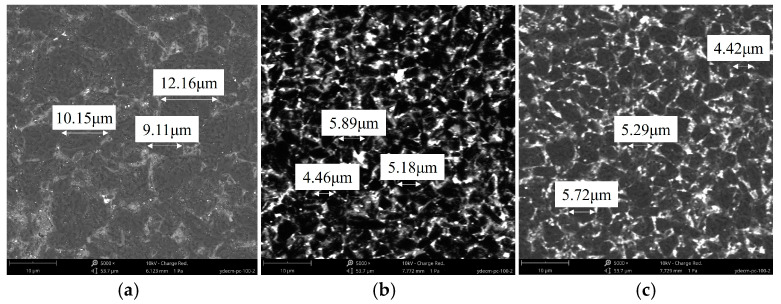
Different material microstructures of PCBN tools. (**a**) Material A with larger particles; (**b**) Material B; (**c**) Material C with a lower Al percentage.

In this work, three PCBN materials with different microstructures were used to prepare the PCBN tools, as depicted in Figure 2. The main distinction in material microstructure between PCBN materials A and B is particle size. Their particle sizes are 8~12 μm and 4~6 μm, corresponding to PCBN materials of large particles and fine particles, respectively. The distinction in microstructure between the PCBN materials B and C is their adhesive phases. The adhesive phases in PCBN usually include Al, TiC, and WC. During the adhesive phase, the percentage of Al-Ti has a significant effect on PCBN material performance. In this paper, the Al percentage in the adhesive phase of PCBN materials B and C are 17% and 12%, respectively.

**Figure 3:**
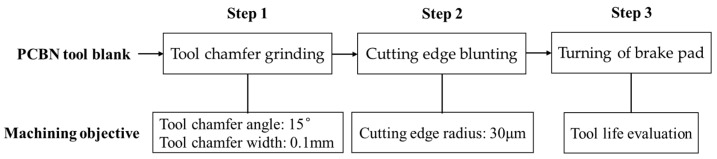
The flow diagram of tool edge preparation.

**Figure 4:**
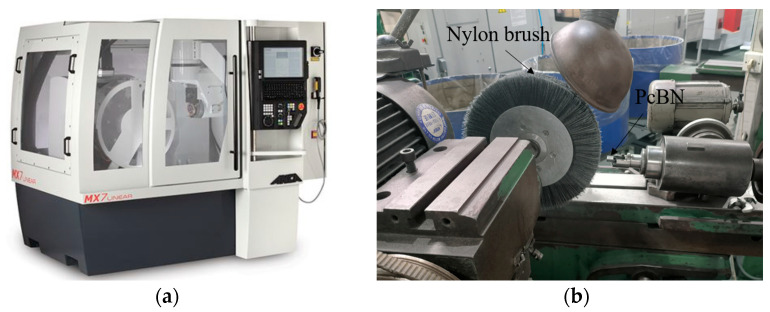
The cutting edge preparation experiment of the PCBN tool. (**a**) Tool chamfer grinding experiments; (**b**) Tool edge blunting experiments.

The flow diagram of tool edge preparation in this work is exhibited in Figure 3. In order to prepare the cutting edge of PCBN tools, the grinding process was used to machine the tool chamfer width and angle, and a subsequent blunting process was used to machine the edge round. In the first step, the tool chamfer grinding experiments were conducted with the tool grinder MX7 Linear (ANCA group, Melbourne, Australia), as depicted in Figure 4a. In the grinding process, a diamond wheel was used, and the tool chamfer was machined with the fixed grinding depth and path based on the target chamfer width and angle. As depicted in Table 1, the grinding depth was fixed to 2 μm, the grinding velocity was set to five levels in the range of 18~30 m/s, and the feed rate was set to five levels in the range of 40~160 mm/min. The optimal grinding parameters for the tool chamfer were obtained in this step based on the machining accuracy of the tool chamfer.

In the second step, an edge blunting experiment was conducted with the tool grinder M6025H (Omikron Machine Tool Co., Ltd, Wuhan, China), as depicted in Figure 4b. The nylon brush blunting method was used. Micro abrasive media are coated onto a brush wheel that is made of nylon material, and then the brush wheel is installed on the spindle of the tool grinder. The flexible brush wheel rotates to passivate the tool edge with the coated abrasives. In the tool edge blunting process, the rotation speed of the brush wheel is fixed to 60 rpm. The blunting time varies and optimizes in the range of 17~33 s to obtain different edge radii, according to the preliminary experimental results. The optimal blunting time for the cutting edge is obtained in this step. All the experiments were repeated three times, and the average value was adopted as the result in this paper.

After tool edge preparation of the PCBN tool, a laser microscope VK-X3000 (Keyence, Osaka, Japan), made by the Keyence group, was used to inspect the tool chamfer parameters, the edge radius, and the surface roughness on the flank face. All the measurements were carried out in triplicate, and their average values were used for the experiment results. Among them, the machining error between the target and the measured chamfer parameters was adopted to evaluate the edge preparation quality, such as the chamfer width and angle.

**Figure 5:**
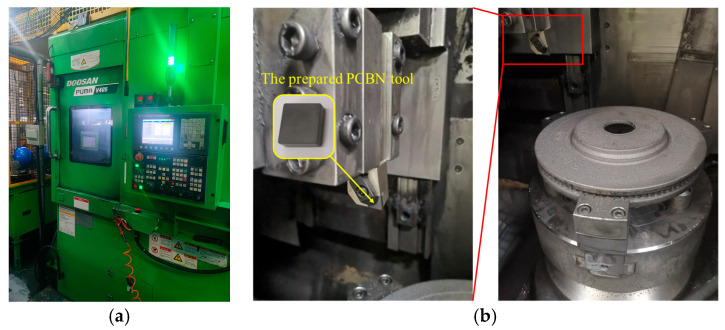
The prepared PCBN tool test in the machining of brake pads. (**a**) The vertical turning center; (**b**) The turning of the brake pad.

In the third step, the prepared PCBN tools were verified in the turning of the brake pad, as shown in Figure 5. In the turning test, a brake pad made of gray cast iron F220P was used as a workpiece. The vertical turning center PUMA V405 (DN Solutions, Shandong, China), was used in the test. The tool was installed in the indexable tool turret with the tool holder TRLNL2525M12 (ZCCCT, Zhuzhou, China). A semi-synthetic milk-based cutting fluid was used. Based on the previous research, the machining speed was set to 500 m/min, the feed rate was 0.5 mm/r, and the cutting depth was 3 mm. The tool life is counted by the number of machined workpieces with the required surface roughness standard of 0.5 μm and a flatness tolerance of 0.1 mm to evaluate the tool performance.

## 3. Results and Discussion

### 3.1. The Effect of Material Microstructures on Chamfer Grinding of the PCBN Tool

**Figure 6:**
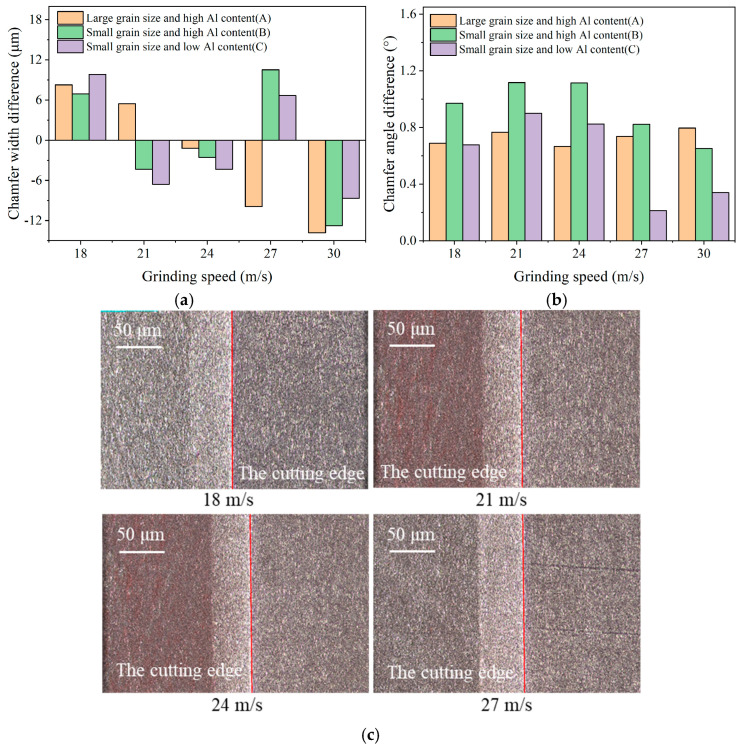
Tool chamfer angle and width error under different grinding speeds. (**a**) Chamfer angle error; (**b**) Chamfer width error; (**c**) Tool chamfer morphology (Material B).

In the tool chamfer grinding, the grinding velocity and feed rate were varied in five levels to analyze the effect of material microstructures and optimize the grinding parameters. After the tool chamfer grinding, the tool chamfer angle and width error were calculated by subtracting the measured value from the target value. The tool chamfer width and angle error under different grinding velocities are depicted in Figure 6. From Figure 6a, the machined chamfer angles of the three materials are all larger than the target value of 15°, which exhibits a positive error. In the grinding process, the diamond wheel has an inevitable runout tolerance, resulting in a tool chamfer angle larger than the target value. With an increase in grinding speed from 18 m/s to 30 m/s, materials A and B exhibit less variation in tool chamfer angle error, but material C exhibits a gradual reducing trend in tool chamfer angle error. From Figure 6b, the tool chamfer width is larger than the target value of 0.1 mm under the low grinding speed, and becomes less than the target value under the high grinding speed. In terms of the absolute value of the tool chamfer width error, it exhibits a decreasing trend at first, and then a reverse increasing trend with the increase in the grinding speed. The tool chamfer morphology under different grinding speeds is depicted in Figure 6c. The cutting capacity of the diamond grains on the wheel is relatively poor under a low grinding speed of 18 m/s, and it is difficult to cut off CBN particles during the grinding process. Some CBN particles will pull out or peel off from the tool chamfer edge, resulting in a larger tool chamfer width error. However, the increased runout tolerance with a grinding speed higher than 27 m/s also leads to a larger tool chamfer width error. Hence, the smallest tool chamfer width error is achieved under the grinding speed of 24 m/s, which presents smooth and sharp tool edge morphology. Overall, the results show that a tool chamfer angle error ≤ 1° and a tool chamfer width error ≤ 10 μm can be obtained under the appropriate machining parameters, which completely meet the M-level precision requirements of a PCBN tool with the grade CNMN120412.

**Figure 7:**
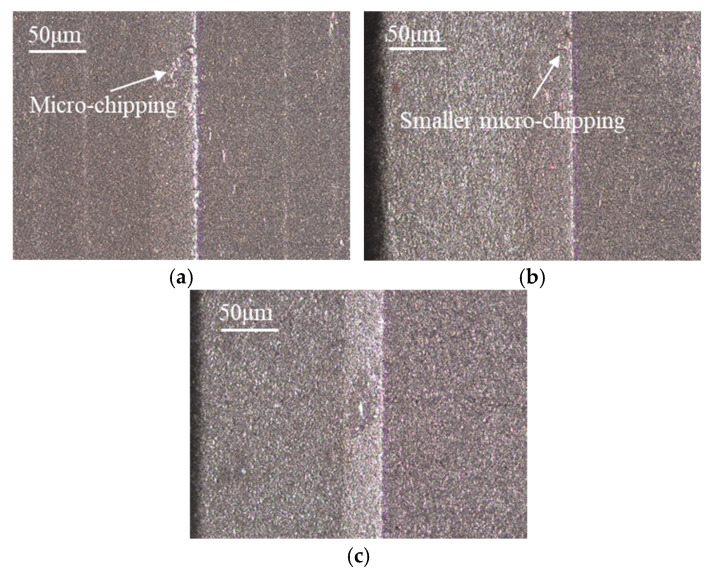
Tool chamfer morphology with different material microstructures (*v* = 27 m/s). (**a**) Material A; (**b**) Material B; (**c**) Material C.

By calculating the averaged error values of three materials, the obtained tool chamfer angle errors are 0.73°, 0.93°, and 0.59°; the obtained tool chamfer width errors are 7.73 μm, 7.42 μm, and 7.21 μm, respectively. In comparison, it is found that the tool chamfer width error of material A with a larger particle size is slightly bigger than that of material B with a fine particle size, although its tool chamfer angle error is smaller. By comparison, both the tool chamfer angle and the width error of material B with higher Al content are larger than those of material C. This indicates that the PCBN material with a higher Al content ususally exhibits worsening chamfer quality in tool edge grinding. Tool chamfer morphology with different material microstructures is depicted in Figure 7, which is consistent with the average error of tool chamfer parameters. It can be observed that there is some micro-chipping on the tool edge of material A made with a large particle size, which presents the largest tool chamfer width error. Smaller micro-chipping can also be found on the tool edge of material B with higher Al content. The tool edge of material C with fine particle size and lower Al percentage is the most smooth and sharp.

From the material microstructures in Figure 2, PCBN materials with a larger particle size present high hardness, and also a larger contact area with the diamond particles during the grinding process [21,22]. This leads to greater friction behavior and higher grinding forces on PCBN particles, which easily causes the breaking of CBN particles and micro-chipping on the cutting edge, resulting in a larger tool chamfer error of material A. In material with higher Al content, the hardness decreases, and the binder phase is prone to local agglomeration, thereby reducing the bonding strength between CBN particles. CBN particles with lower bonding strength are easily pulled out during grinding, and the induced pull-out pits further increase the tool chamfer error of material B.

**Figure 8:**
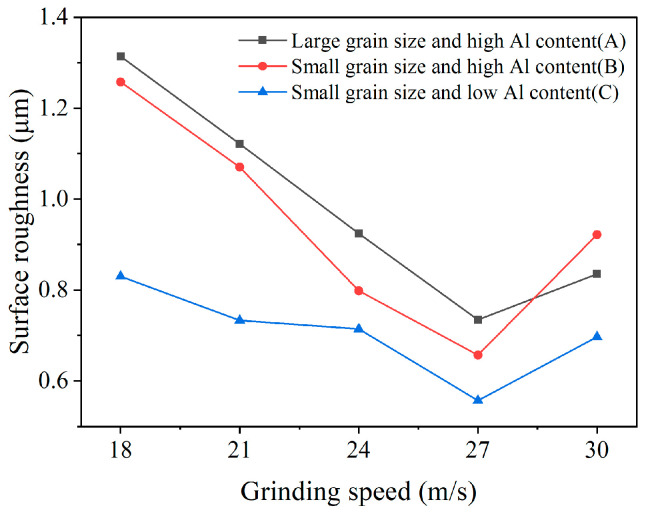
Flank surface roughness of PCBN tool under different grinding speeds.

**Figure 9:**
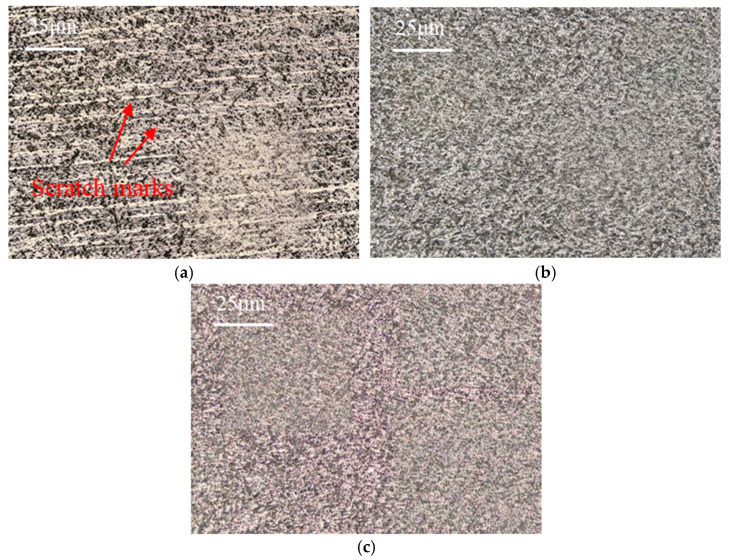
Flank surface morphologies with different material microstructures (*v* = 27 m/s). (**a**) Material A; (**b**) Material B; (**c**) Material C.

Surface roughness on the tool flank face under different grinding speeds is presented in Figure 8. As the grinding speed increases from 18 m/s to 30 m/s, surface roughness first exhibits a decreasing trend and then reverses to an increasing trend; the minimum roughness is attained at a grinding speed of 27 m/s. A comparison among PCBN materials with different microstructures reveals a trend similar to that observed for tool chamfer width error. Material A displays the highest surface roughness, followed by material B, while material C achieves the lowest roughness, with an average value of 0.99 μm, 0.94 μm, and 0.71 μm, respectively. The variation in surface roughness is closely related to material microstructure. Due to higher material hardness and low fracture toughness, PCBN materials composed of larger particles are more susceptible to grain pull-out or breakage during grinding, which in turn increases tool surface roughness. As shown in Figure 9a, some scratch marks caused by the pulled grains are visible on the tool face of material A. For material B, the higher Al content may promote local agglomeration of the binder phase, thereby facilitating localized pull-out of CBN particles during grinding. The pulled pits lead to a comparatively higher tool surface roughness, as depicted in Figure 9b. Material C possesses an appropriate binder phase that can decrease grain pull-out by the mutual constraint between CBN particles, and obtains the smoothest surface morphology, shown in Figure 9c.

From the results, it is found that the optimal grinding speed for the tool chamfer and surface roughness is not consistent. In this paper, comprehensive normalization analysis is used to determine which parameter is optimal. Firstly, the normalization indicator $Q_{i}$ of chamfer angle and width error, and the surface roughness was, respectively, calculated by Formula (1), and then their total normalization indicator $Q_{total}$ was calculated by Formula (2):

(1) $$Q_{i}=(X_{i}-X_{min})/(X_{max}-X_{min})$$

(2) $$Q_{total}=\sum_{1}^{n}\left(Q_{i}\right)$$

**Figure 10:**
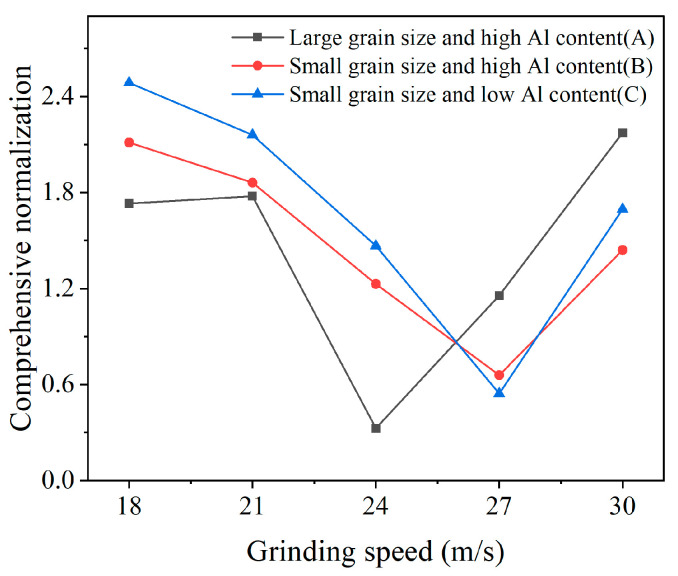
The cutting speed optimization based on the comprehensive normalization.

The calculated normalization indicator $Q_{total}$ is exhibited in Figure 10. It is found that the comprehensive normalization indicator of the three materials all exhibit a decreasing trend at first, and then show a reverse rising trend with an increase in the grinding speed. The lowest comprehensive normalization indicator is achieved under the grinding speed of 24 m/s for PCBN material A, and 27 m/s for PCBN materials B and C. This indicates that PCBN material A with a large grain size should be machined at a relatively low grinding speed, and the PCBN material with a fine grain size can be machined at a higher grinding speed.

**Figure 11:**
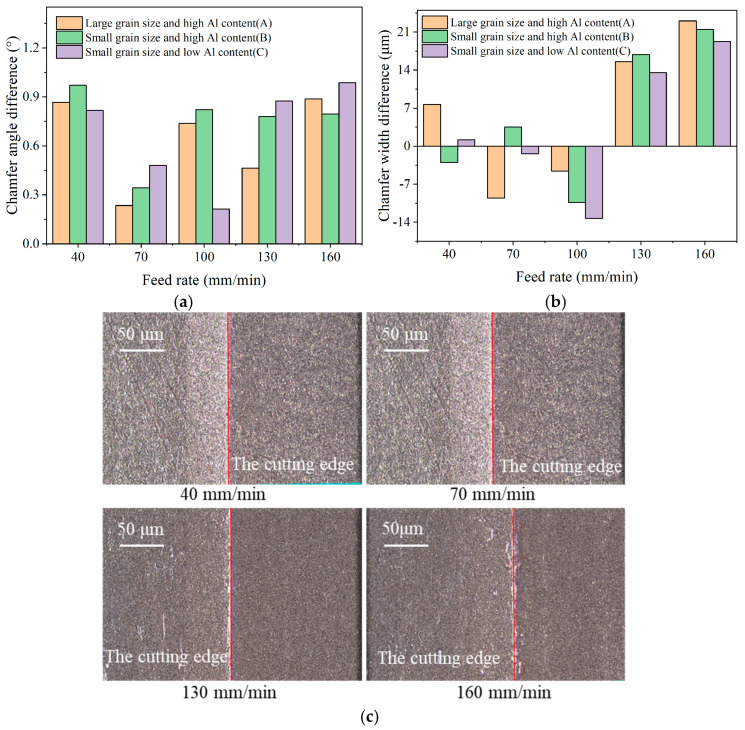
Tool chamfer width and angle error under different feed rates. (**a**) Tool chamfer angle error; (**b**) Tool chamfer width error; (**c**) Tool chamfer morphology (Material C).

Figure 11 presents the tool chamfer angle error and chamfer width error under different feed rates. As shown in Figure 11a, when the feed rate increases from 40 mm/min to 160 mm/min, the tool chamfer angle error initially decreases and then exhibits a reverse increasing trend. The minimum angle error is attained at 70 mm/min for materials A and B, and at 100 mm/min for material C. All the tool chamfer angle errors remain within 1°, which fully satisfies the M-level precision requirements. The tool chamfer width error is depicted in Figure 11b. From the results, at low feed rates, the tool chamfer width is smaller than the target value of 0.1 mm, whereas it becomes larger than the target value when the feed rate exceeds 100 mm/min. The absolute value of tool chamfer width errors of the three materials all display a gradual increasing trend with the feed rate, reaching the maximum values of 23.05 μm, 21.50 μm, and 19.28 μm for materials A, B, and C, respectively. These maximum errors clearly exceed the allowable tolerance for M-level precision. Only at feed rates below 100 mm/min, the tool chamfer width error remains within ±10 μm, thereby meeting the M-level precision requirements.

Tool chamfer morphologies obtained at different feed rates are shown in Figure 11c. At feed rates of 70 mm/min and 100 mm/min, the tool chamfer surface is smooth and continuous, exhibiting a distinct edge boundary and remaining free of micro-chipping or other defects. When the feed rate is increased to 130 mm/min, noticeable micro-chipping begins to appear along the tool edge boundary, resulting in a discontinuous boundary. This phenomenon becomes more severe at 160 mm/min. At excessively high feed rates, diamond abrasives cannot effectively cut off CBN particles; instead, CBN particles located at the tool edge are prone to pull-out or break under the relatively large grinding forces, thereby generating micro-chipping at the tool chamfer boundary adjacent to the cutting edge.

**Figure 12:**
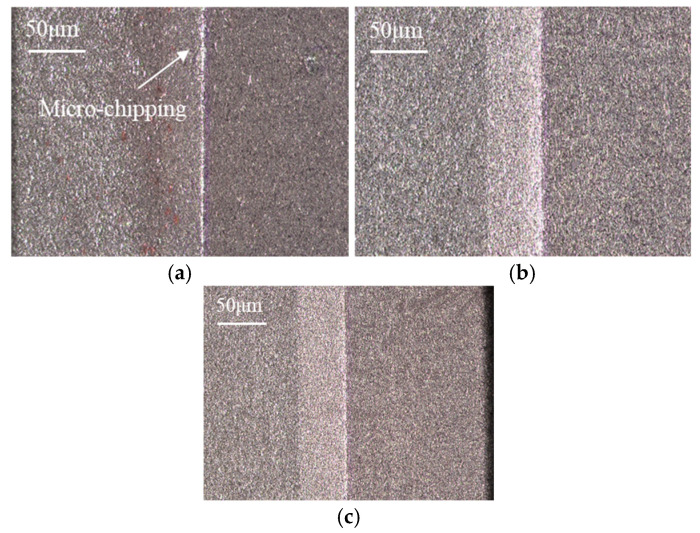
Tool chamfer morphology with different material microstructures (*f* = 70 mm/min). (**a**) Material A; (**b**) Material B; (**c**) Material C.

The average tool chamfer angle errors for the three materials are 0.64°, 0.74°, and 0.67°, and the average tool chamfer width errors are 12.08 μm, 11.03 μm, and 9.74 μm, respectively. Combined with the tool chamfer morphologies shown in Figure 12, the following comparisons can be drawn. With regard to the effect of particle size, material A, which possesses a larger particle size, exhibits a higher tool chamfer width error than material B, and smaller micro-chipping can be observed along its tool chamfer edge. This indicates that coarser CBN particles are more prone to incur edge damage during grinding, leading to increased dimensional deviation. With regard to the effect of the binder phase, material B, which has a higher Al content, obtains both higher tool chamfer width and angle errors compared with material C. Furthermore, the tool chamfer edge boundary of material C is visibly sharper and more uniform than that of material B, indicating that an appropriate binder phase can effectively improve the dimensional accuracy and edge quality of the tool chamfer.

**Figure 13:**
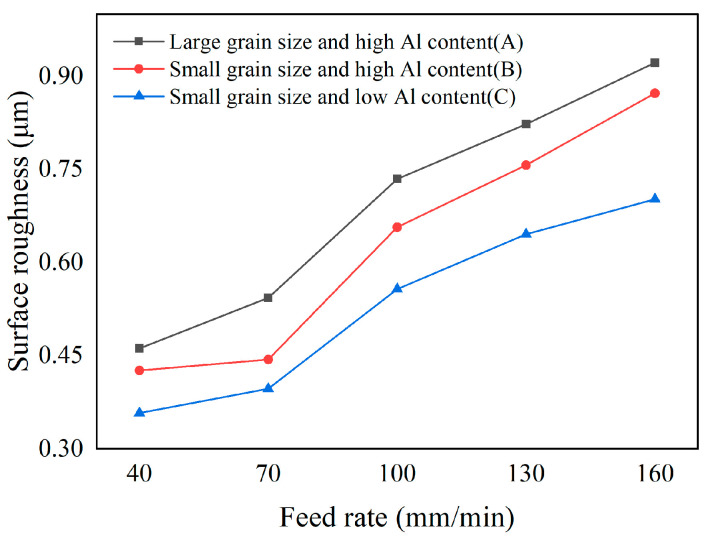
Flank surface roughness of PCBN tool under different feed rates.

The flank face surface roughness measured at different feed rates is presented in Figure 13. As the feed rate increases from 40 to 160 mm/min, the surface roughness of the three materials exhibits an increasing trend. Surface roughness rises from 0.46 μm, 0.43 μm, and 0.36 μm to 0.92 μm, 0.87 μm, and 0.70 μm, corresponding to an increase of 99.8%, 104.8%, and 96.2%, respectively. This result can be attributed to the increase in the undeformed chip thickness of single diamond abrasives at higher feed rates, combined with a shorter contact time between the workpiece and diamond grains, which promotes a rougher ground surface [21]. In comparison, the averaged surface roughness of the three tools with different material microstructures is 0.69 μm, 0.63 μm, and 0.53 μm. It is found that material A consistently exhibits the highest roughness, material B is intermediate, and material C obtains the lowest value. This result is consistent with tool chamfer width error results, further confirming that the material microstructure plays a critical role in determining the ground surface quality.

**Figure 14:**
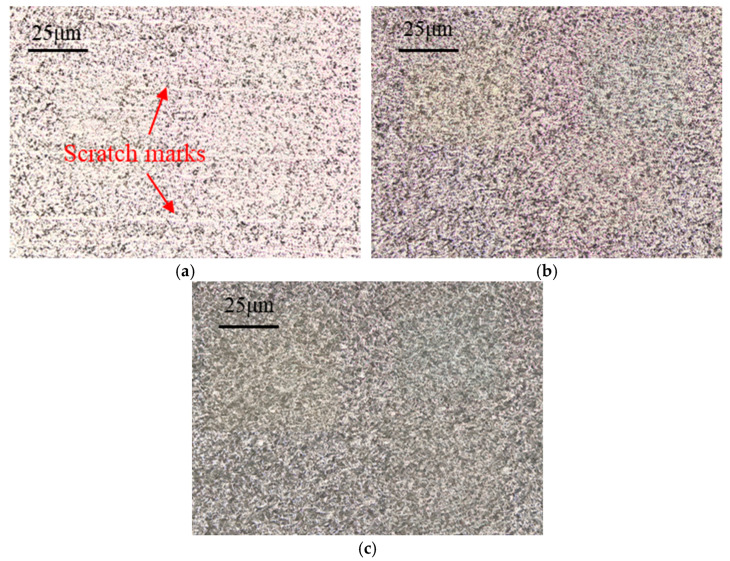
Tool surface morphology with different material microstructures (*f* = 70 mm/min). (**a**) Material A; (**b**) Material B; (**c**) Material C.

The surface morphologies of the three materials ground at a feed rate of 70 mm/min are compared in Figure 14. Numerous micro scratch marks are clearly visible on the tool surface of material A, which possesses the coarsest CBN particles. These scratches are most likely caused by the pulled particles being dragged across the tool surface, leading to a significant degradation of the surface finish. In contrast, materials B and C with fine particles are free of such scratch marks, exhibiting a more uniform and smooth tool surface morphology, which accounts for their relatively low surface roughness. In particular, material C, with the suitable binder phase, achieves the most favorable tool surface quality.

**Figure 15:**
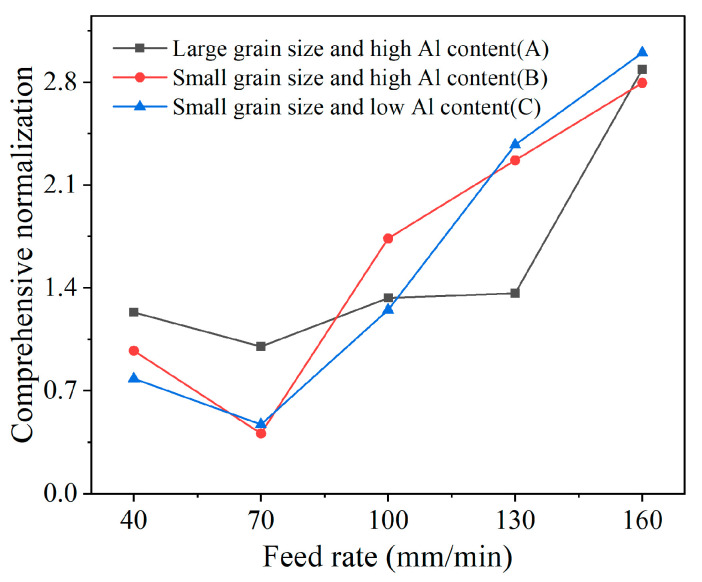
The feed rate optimization based on comprehensive normalization.

To acquire the optimal feed rate that synthetically considers all tool chamfer quality parameters, a comprehensive normalization analysis was also performed based on Formulas (1) and (2). The comprehensive normalization value $Q_{total}$ that was calculated from the tool chamfer angle and width error, and surface roughness, is shown in Figure 15. From the results, the lowest comprehensive normalization indicator can be acquired under the feed rate of 70 mm/min for all three PCBN tools, which is the optimal machining parameter to obtain better edge quality.

### 3.2. The Effect of Material Microstructures on Tool Edge Blunting of the PCBN Tool

**Figure 16:**
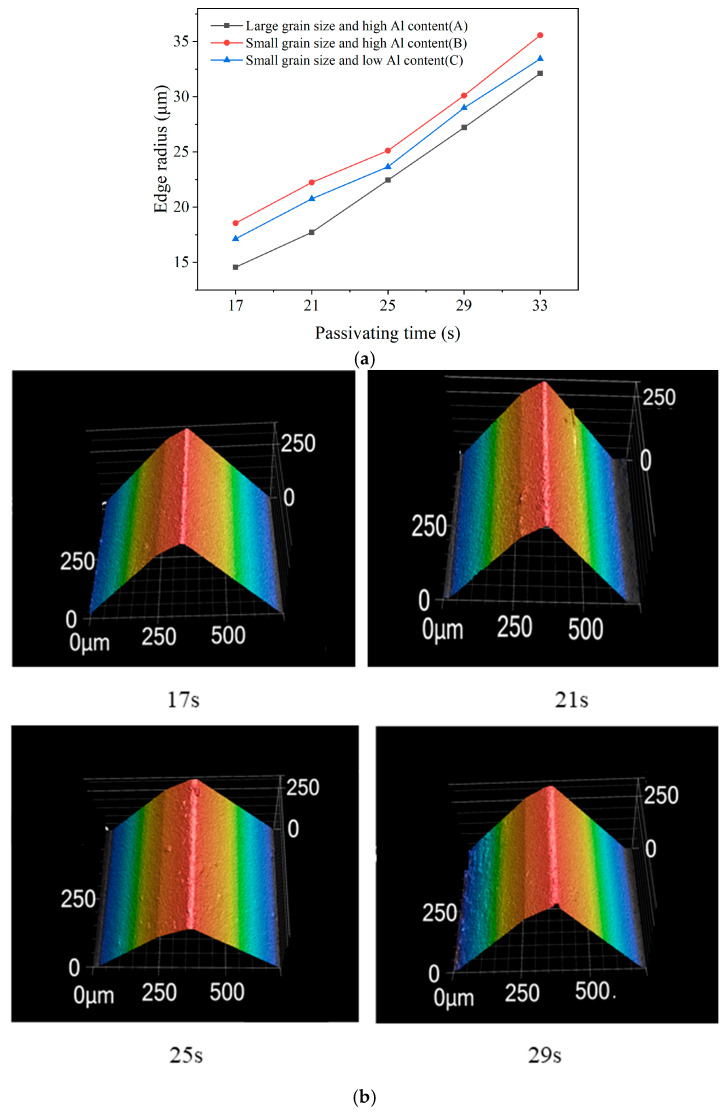
Tool edge radius and morphology under different blunting times. (**a**) Tool edge radius; (**b**) Tool edge morphology (Material B).

After the tool chamfer grinding process, tool edge blunting was performed on the PCBN tools to enhance edge strength. In this operation, a flexible brush wheel continuously brushes the cutting edge to produce a rounded and smooth edge profile. Prior to blunting, the initial edge radius is approximately 10 μm. The variation of the tool edge radius with different blunting times is shown in Figure 16a. The tool edge radius increases progressively with the blunting time, reaching 32.12 μm, 35.57 μm, and 33.43 μm for the three materials after 33 s of blunting time. This trend indicates that a blunting time of approximately 30 s is optimal for achieving the target edge radius of 30 μm. Figure 16b presents the tool edge morphologies of material B at different blunting times. At a blunting time of 17 s, the cutting edge remains very sharp, the rounded arc profile is barely visible, and some micro-defects persist along the edge. As blunting time increases, the rounded arc profile becomes more pronounced, and micro-defects are progressively removed, resulting in a smoother and more uniform edge.

**Figure 17:**
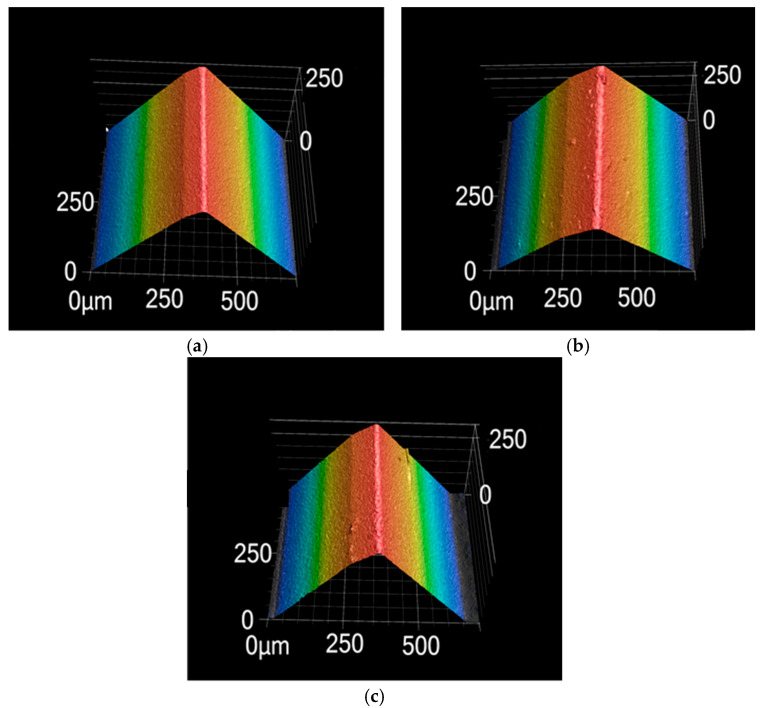
Tool edge morphology with different microstructures (blunting time *t* = 25 s). (**a**) Material A; (**b**) Material B; (**c**) Material C.

Based on the results in Figure 16a, under the same blunting conditions of 25 s, the average edge radii of materials A, B, and C are 24.88 μm, 28.26 μm, and 26.71 μm, respectively. Among the three materials, material A exhibits the smallest edge radius after blunting, material C obtains an intermediate value, and material B obtains the largest. Tool edge morphologies with different material microstructures after identical blunting time are compared in Figure 17. With regard to the effect of CBN particle size, the rounded arc profile of the cutting edge of material A is visibly smaller than that of material B. This indicates that material A, with its larger CBN grains and higher material hardness, is more resistant to material removal during the brushing process and, therefore, more difficult to blunt, generally requiring a slightly longer blunting time to reach the same tool edge radius. Regarding the effect of the binder phase, the rounded arc profile of material C is also smaller than that of material B. This suggests that material C, which possesses a lower Al content and, consequently, a higher intrinsic hardness, is likewise more difficult to blunt, as its harder binder phase resists the abrasive action of the brush wheel more effectively. Material B, with a smaller particle size and higher Al content, is the most prone to blunt among the three materials, due to its lowest hardness, which is the critical factor during tool edge blunting.

### 3.3. The Application of PCBN Tools in the Turning of Brake Pads

**Figure 18:**
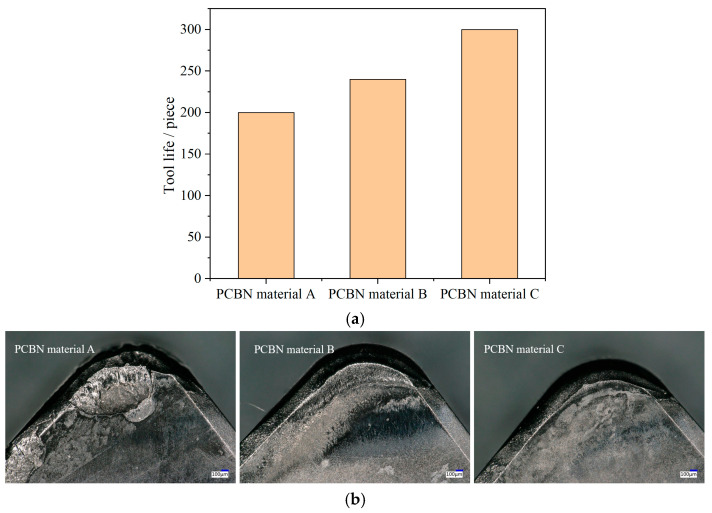
Tool life and tool wear of PCBN tools with different material microstructures. (**a**) Tool life; (**b**) Tool wear morphology.

After the tool edges were prepared using the optimized chamfer grinding and edge blunting parameters, the PCBN tools were applied in the turning of brake discs made of gray cast iron F220P, a material widely used in Japanese automobiles. On the factory production line, tool life was evaluated by the number of workpieces machined while maintaining the specified surface roughness and flatness tolerance. Tool life and wear morphologies of three PCBN tools with different material microstructures are shown in Figure 18. From the results, the tool life per each cutting edge reached 200, 240, and 300 workpieces for materials A, B, and C, respectively.

A comparison of materials with different grain sizes reveals that material B, with its finer CBN grains, achieves a significantly longer tool life than the coarser-grained material A. Correspondingly, the worn rake face of material A exhibits pronounced edge chipping, whereas material B predominantly displays typical crater wear characteristics, as shown in Figure 18b. This difference suggests that the larger grains in material A are more susceptible to fracture under the cyclic mechanical and thermal loads of machining, leading to a rapid loss of cutting edge integrity due to its low fracture toughness. In contrast, the fine-grained structure of material B provides higher toughness and better resistance to edge chipping, promoting a more gradual and stable wear mode. Regarding the effect of the binder phase, the wear patterns of materials B and C are similar. However, material C, which contains a lower Al percentage and, consequently, possesses a higher material hardness, exhibits a visibly smaller wear zone than material B. This observation demonstrates that the suitable binder phase enhances the material’s resistance to tool wear.

## 4. Conclusions

In this work, tool chamfer grinding and edge blunting were performed on three PCBN tools with different material microstructures, and the prepared PCBN tools were verified in the turning of brake pads. According to the experimental results, the following conclusions were acquired.

1. As the grinding speed increases, both the tool chamfer width error and the flank face surface roughness first decrease and then exhibit a reverse increasing trend. The optimal grinding speed is 24 m/s for the PCBN material with coarse grains and 27 m/s for the fine-grained material. As the feed rate increases, tool chamfer width error and surface roughness rise gradually, while tool chamfer angle error initially decreases before increasing; the optimal feed rate is determined to be 70 mm/min. Among different material microstructures, the PCBN material with coarser grains obtains a larger tool chamfer width error and higher surface roughness compared with the fine-grained material. Similarly, the material with a higher Al content in the binder phase produces greater tool chamfer width and angle errors as well as higher surface roughness than the material with a lower Al content.
2. The PCBN material with a larger grain size is more resistant to material removal and, therefore, more difficult to blunt than the fine-grained material, due to the high hardness. Likewise, the low-Al content material, owing to its higher material hardness, is more difficult to blunt than the high-Al content material. The optimal blunting time for the target edge radius of 30 μm is found to be around 30 s.
3. In the turning of brake discs, the fine-grained PCBN tool achieves a longer tool life than its coarse-grained counterpart. The dominant wear mode of the coarse-grained tool is edge chipping, while the fine-grained tool exhibits predominantly crater wear. The PCBN tool with a lower Al content obtains a longer tool life than that with a higher Al content, despite the similar wear morphologies. Overall, the PCBN tool combining fine CBN grains and low-Al binder phase demonstrates the best performance in terms of edge quality, dimensional accuracy, and tool life in the machining of brake pads.

## Figures and Tables

**Table 1:** Tool chamfer grinding parameters.

Parameters	Value
Grinding depth *a_p_*/μm	2
Grinding velocity *v*/m/s	18, 21, 24, 27, 30
Feed rate *f*/mm/min	40, 70, 100, 130, 160

## Data Availability

All data generated during this study are included in this article.
